# Perisaccadic Remapping and Rescaling of Visual Responses in Macaque Superior Colliculus

**DOI:** 10.1371/journal.pone.0052195

**Published:** 2012-12-17

**Authors:** Jan Churan, Daniel Guitton, Christopher C. Pack

**Affiliations:** Montreal Neurological Institute, McGill University, Montreal, Canada; University College London, United Kingdom

## Abstract

Visual neurons have spatial receptive fields that encode the positions of objects relative to the fovea. Because foveate animals execute frequent saccadic eye movements, this position information is constantly changing, even though the visual world is generally stationary. Interestingly, visual receptive fields in many brain regions have been found to exhibit changes in strength, size, or position around the time of each saccade, and these changes have often been suggested to be involved in the maintenance of perceptual stability. Crucial to the circuitry underlying perisaccadic changes in visual receptive fields is the superior colliculus (SC), a brainstem structure responsible for integrating visual and oculomotor signals. In this work we have studied the time-course of receptive field changes in the SC. We find that the distribution of the latencies of SC responses to stimuli placed outside the fixation receptive field is bimodal: The first mode is comprised of early responses that are temporally locked to the onset of the visual probe stimulus and stronger for probes placed closer to the classical receptive field. We suggest that such responses are therefore consistent with a perisaccadic rescaling, or enhancement, of weak visual responses within a fixed spatial receptive field. The second mode is more similar to the remapping that has been reported in the cortex, as responses are time-locked to saccade onset and stronger for stimuli placed in the postsaccadic receptive field location. We suggest that these two temporal phases of spatial updating may represent different sources of input to the SC.

## Introduction

We tend to perceive a stable visual world, despite frequent eye movements that shift the positions of objects across the retina. One of the mechanisms underlying such visual stability is thought to be a corollary discharge signal [1]–[2] that can be used to predict the perceptual consequences of impending eye movements. Although the existence of a corollary discharge signal has been demonstrated in several studies [3], [4]–[5] (for review e.g. [6]–[7]), the details of its implementation are not well understood.

An interesting example of the influence of corollary discharge signals on visual processing is the *remapping* of visual space that occurs around the time of a saccade. During remapping, the positions of visual receptive fields shift before the start of the saccade to the spatial location they will occupy after the saccade. This mechanism is thought to link the pre-saccadic and post-saccadic retinal images. Remapping has been observed in the lateral intraparietal area (LIP) of monkey cortex [8], [9]–[10]; the superior colliculus [11], [12]–[13], the Frontal Eye-Field (FEF) [14], [15]–[16], and visual areas V4 [17], V3, V3A, and V2 [18].

The corollary discharge signal in FEF depends in part on the ascending pathway from SC through the medio-dorsal nucleus of the thalamus into the FEF. Blocking this pathway at the level of the thalamus reduces remapping in FEF neurons [16] and also reduces trans-saccadic perceptual stability [19]. The source of the remapping responses in the SC is less well understood; they may be generated independently within the SC, or they may be relayed to the SC from cortical areas like FEF or LIP [13]. In order to constrain hypotheses about the circuitry of remapping in the SC, we have performed a detailed analysis of the time-course of the responses to stimuli placed outside the classical receptive fields of individual neurons in the intermediate layers of the SC. We find that these responses can be divided into two categories, based on their timing. At long latencies, they are consistent with the type of remapping typically found in the cortex. In contrast responses that occur at shorter latencies have properties that are consistent with a perisaccadic increase in visual sensitivity. These two types of responses may have different anatomical sources and behavioral functions.

**Figure 1:**
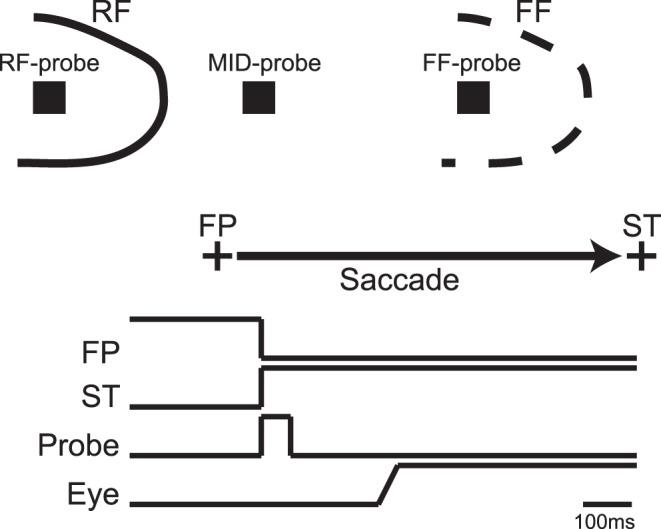
The remapping paradigm. Sketch of the spatial (A) and the temporal (B) properties of the different experimental conditions in the remapping paradigm. A: A visual probe was flashed at different positions in the visual field. Across different, randomly-interleaved conditions, the probe appeared in the receptive field of the neuron (RF-probe), the future field (FF-probe), and at the midpoint between the two (MID-probe). B: The lines represent the relative timing of the fixation point (FP), the saccade target (ST), the remapping probe (Probe) and a typical eye movement (Eye). After the monkeys had fixated for a random duration, the fixation point was extinguished and at the same time the saccade target and the remapping probe appeared. The saccade target remained on for the rest of the trial, while the probe lasted for 59 ms. The monkey made a saccade to the target with a latency that was typically >120 ms; thus the offset of the visual probe and the start of the saccade were separated by at least 60 ms.

## Materials and Methods

### Physiological Procedures

Two adult rhesus monkeys (macaca mulatta) participated in the experiments. Both monkeys had constant access to food as well as to environmental enrichment. Each monkey underwent a sterile surgical procedure to implant a headpost and recording cylinder over the SC as described in detail elsewhere [20]. The physical and psychological health of the animals was monitored by animal care technicians and veterinarians, who provided analgesia and medication as necessary during pre-, intra-, and post-operative time periods. After a post-operative recovery period, the monkeys were seated in a primate chair (Crist Instruments) and trained to maintain fixation and to make visually-guided and delayed saccades towards stimuli presented on a screen. The eye position was recorded by a video eye-tracker (EyeLink 1000, SR Research) for one monkey and by an implanted scleral eye coil [21] for the other monkey; the sampling rate of both systems was 1000 Hz. At the end of the project both monkeys were sacrificed using a painless fatal overdose of veterinary euthanasia solution (Euthanyl, containing 240 mg/ml sodium pentobarbital). All procedures were approved by the Animal Care Committee of the Montreal Neurological Institute, and were in compliance with regulations established by the Canadian Council of Animal Care.

The SC was identified based on an anatomical fMRI scan, as well as the physiological pattern of visual and saccade-related neuronal responses. To obtain a substantial number of neurons from the deeper layers, where neurons with remapping responses were observed to be more frequent [11], in ∼40% of the penetrations we pushed the electrode through all collicular layers until the typical visual and saccade-related activity disappeared, and from this position we started searching for neurons by moving the electrode upwards. Single units were recorded using tungsten microelectrodes (FHC) with a typical impedance of ∼2 MΩ. The signal was sampled at 40 kHz. Single units were identified online and later re-sorted offline using spike sorting software (Plexon Inc.).

**Figure 2:**
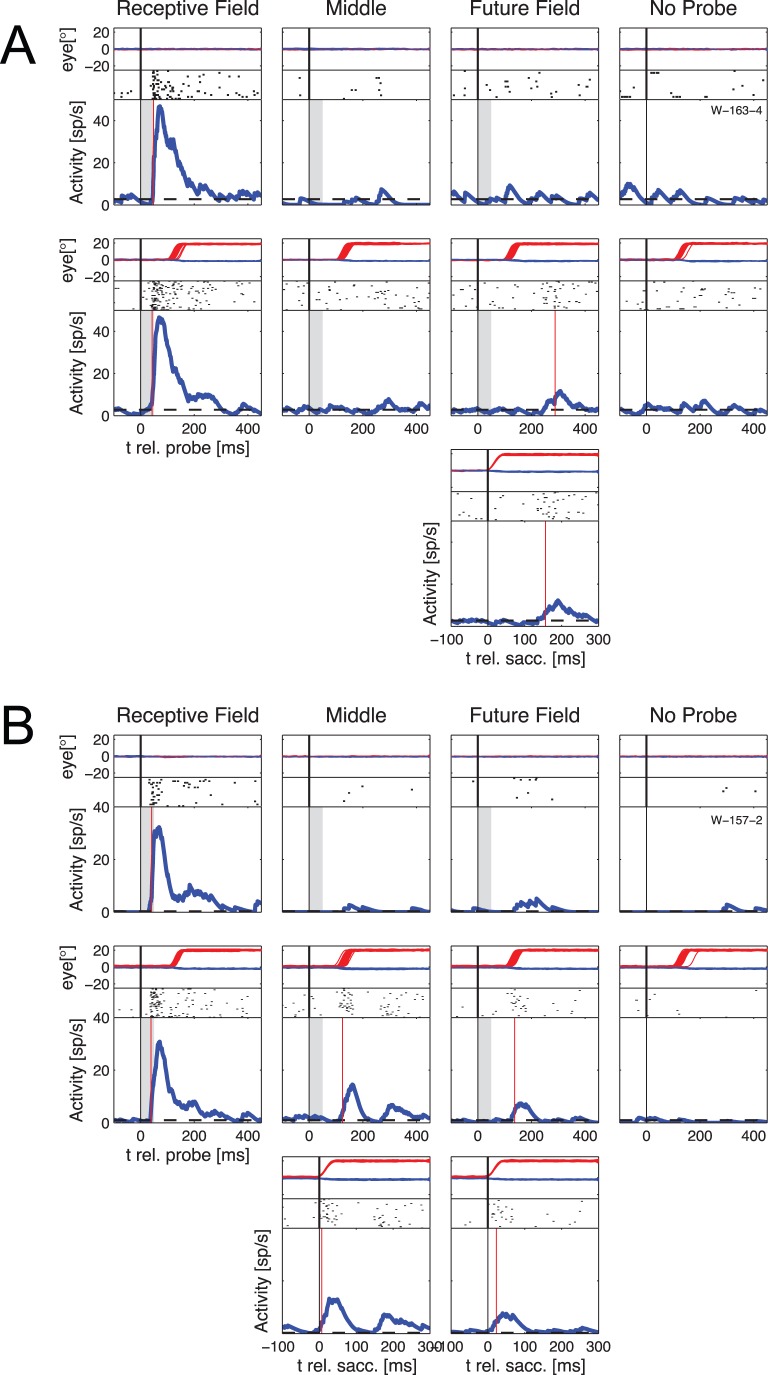
Example of extraclassical responses in single neurons. Eye traces, raster plots and PSTHs obtained during different conditions in two example recordings from visuomotor neurons in the SC. The eye traces are shown in the upper part of each plot (red lines for horizontal, blue lines for vertical). Below are the raster plots and the PSTH. In the PSTH plot the grey area represents the time when the stimulus was presented, and the red vertical line represents the calculated latency of the response (the time at which the response was significantly higher than the baseline activity for the first time). The first row of plots in A and B shows responses to stimuli presented during continuous fixation. The second row shows data obtained when the monkey made visually guided horizontal saccades (amplitude 20°) into the ipsilateral visual hemifield. The third row shows the same responses as the second row aligned to the onset of the saccade for those conditions in which significant extraclassical responses were found. A: Example neuron shows extraclassical activity for probes flashed at the FF position. B: Example neuron shows extraclassical responses for probes flashed at the MID and FF positions.

**Figure 3:**
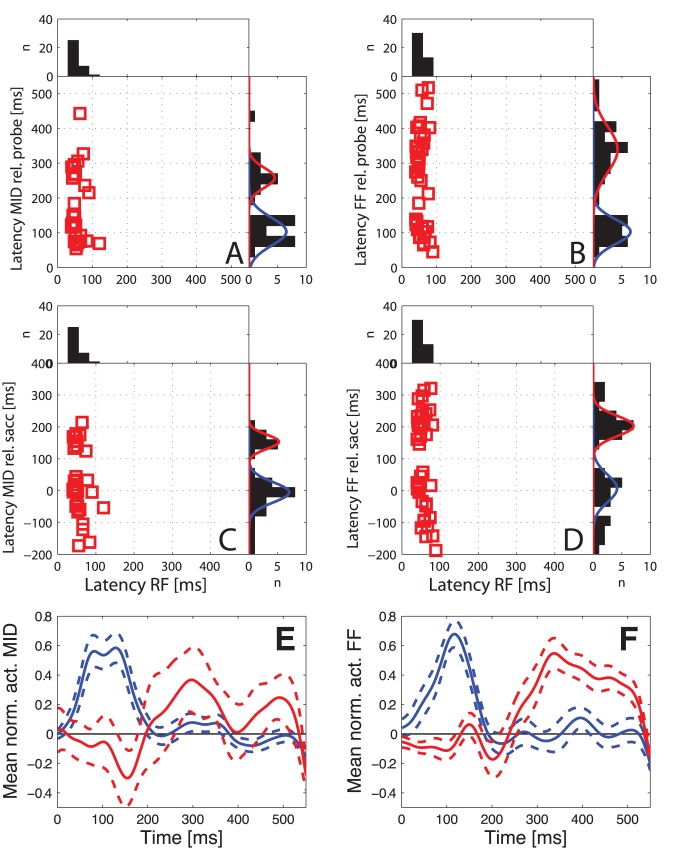
Latencies of visual and extraclassical responses. Latencies of the responses to probes presented in the RF, MID and FF positions across the two monkeys. The latencies for the RF-probes are always shown relative to the onset of the probe, while responses to the MID- and FF-probes are shown relative to the onset of the probe (A and B) or relative to saccade onset (C and D). Histograms aligned to each axis show the distributions of latencies on this axis. A sum of two Gaussians was fitted to the distribution of remapping latencies. The two resulting Gaussians are plotted in blue for early responses and red for late responses. E, F: Averaged normalized activities of early responding neurons (blue) and late responding neurons (red) to MID-probes (E) and FF-probes (F). The normalization was done by first subtracting the baseline activity and then dividing by the maximum value. Timing of the responses is shown relative to the onset of the probe. Dashed lines show mean ±1SEM.

### Stimulus Presentation

The stimuli were generated using a standard Pentium III PC computer at a spatial resolution of 800×600 pixels and a presentation frame rate of 85 Hz. The frames were programmed in Matlab v7.0 using the Psychophysics Toolbox [22]–[23] and back-projected on a semi-transparent screen by a CRT video projector (Electrohome 8000). The screen covered an area of 80×50 degrees of visual angle at a viewing distance of 78 cm.

In each paradigm the monkeys were required to keep fixation within ±2.5 deg of the fixation point or saccade target to obtain a small amount of water or juice on each trial.

#### Remapping task

The experiments took place in a dark room, using visual stimuli presented on an otherwise dark screen (background luminance <<0.01 cd/m^2^). This condition has been shown to maximize the incidence of remapping responses in the SC [12]. The task we used to probe spatial remapping around the time of saccades (Figure 1) was similar to a task used for the same purpose by Walker et al. [11]. On each trial monkeys acquired fixation, after which a saccade target was presented in the ipsilateral visual field (relative to the recording site) at a horizontal distance of 20° from the fixation point. At the same time a visual probe (square size 30′, luminance 29 cd/m^2^) was flashed for 59 ms (5 frames). The probe was presented in one of three positions (Figure 1):

1. in the visual receptive field of a neuron (RF-condition) at a position that elicited strong visual responses during fixation
2. in the future field (FF-condition), which was the RF position shifted by the vector of the saccade
3. at the midpoint between the two (MID-condition).

The spatial position of the FF probes was in all cases in the ipsilateral visual hemifield, which constitutes the ‘across hemisphere’ remapping condition [13]. Depending on the eccentricity of the RF under study, the probes in the MID-condition could be located in the ipsilateral visual hemifield, the contralateral visual hemifield, or on the vertical meridian between the two. For later interpretation it is important to note that while the MID probes were presented outside the classical RF, they were in some cases within the future field area (depending on the size and shape of the visual RF). However, in these cases the position of the MID probe would be more in the periphery of the future field than the position of the FF probe and therefore would be expected to elicit weaker remapping responses if remapping were spatially accurate.

**Table 1:** Distribution of latencies (in ms) for early and late extraclassical responses to probes presented at MID and FF positions relative to the onset of the probe and relative to the saccade-onset.

	Relative to saccade onset				Relative to probe onset					
	MID-position		FF-position		MID-position			FF-position		
	Mean	Std	Mean	Std	Mean	Std	n	Mean	Std	n
Early	−5	32	5	37	103	40	21	103	35	18
Late	153	22	202	34	258	28	12	340	66	23

Sample sizes of neurons assigned to early and late remapping based on latencies relative to the onset of the probe.

**Figure 4:**
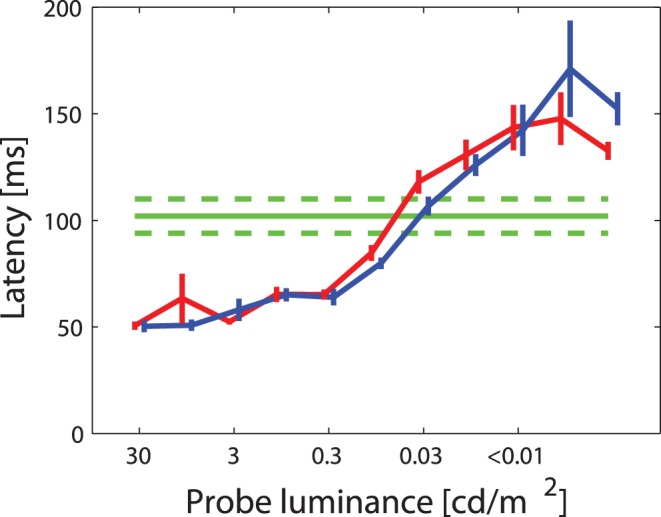
Latencies of visual responses to probes of different luminance. Average mean and SEM of response latencies to probes of different luminance flashed for 59 ms in the RF during fixation (blue) and before saccades (red) compared to the average latency of early remapping responses to FF probes (green line, dashed lines indicate ±1 SEM). The latencies of early extraclassical responses to MID probes were very similar to those of FF probes (Table 1) and are therefore not shown in this figure.

#### Control tasks: fixation and saccade task

In addition to the remapping paradigm, we ran several control experiments to verify that the responses to FF probes and MID probes were neither purely visual nor purely motor. In one type of control trial the visual probes were presented at the three positions during continuous fixation; in another control condition saccades were made to the saccade target without a visual probe. All saccade conditions were randomly interleaved, and at least ∼15 trials were recorded for each condition.

#### Measurement of visual latencies for different probe luminances

To compare the average latencies of responses to MID and FF probes with those of regular visual responses, we flashed probes in the RFs of 48 of the neurons that were also tested in the remapping task. The general procedure, as well as the timing, the position and the size of the stimuli were the same as in the RF-condition of the remapping task. The only difference with respect to the remapping task was the luminance of the visual probes, which varied between 30 cd/m^2^ (the luminance of the probes in the remapping task) and ∼0.005 cd/m^2^. The monkeys were required either to maintain fixation or to make visually guided saccades with the same vector as that used in the remapping task. The fixation and the saccade condition were recorded in separate blocks, but the order of luminances was randomized within each block. At least 10 successful trials were collected for each luminance in the fixation and saccade conditions.

**Figure 5:**
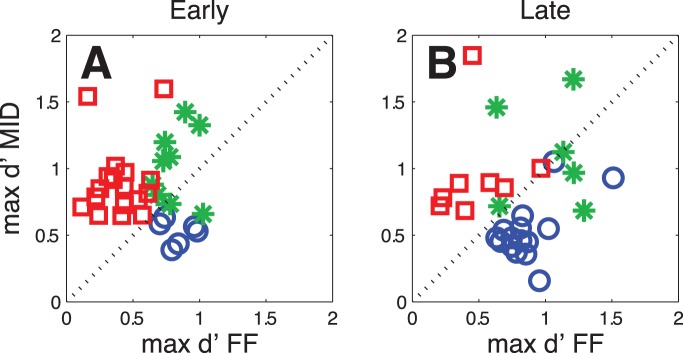
Strength of extraclassical responses. Comparison of the maxima of the remapping responses to MID and FF probes shown separately for early (A) and late (B) extraclassical responses across the two monkeys. Neurons showing significant extraclassical responses only to the MID-probes are marked as red squares, those showing only remapping of the FF-probes as blue circles and those for both probes as green stars.

**Figure 6:**
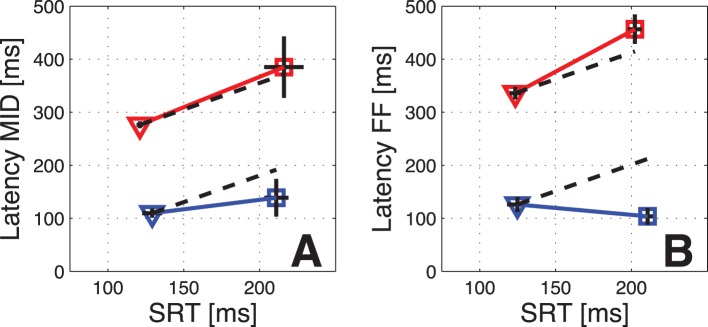
Relationship between saccadic reaction times and extraclassical response latencies. Comparison between the mean saccadic reaction times (SRTs) and the mean latencies of the early (blue) and the late (red) extraclassical responses to MID-probes (A) and FF-probes (B). The latencies are calculated relative to the onset of the visual probe. The results for the two monkeys are shown separately. Monkey 2 (squares) had on average ∼80 ms longer SRTs than monkey 1 (triangles). Black horizontal and vertical lines placed in the markers indicate the SEM of the SRTs and of the neuronal latencies. Dashed black lines show the expected increase in remapping latency that would result from shifting the extraclassical response latency on each trial by an amount equal to the mean SRT.

#### Delayed saccade task

To classify the visual and motor responses of the neurons and to determine the extent and location of their visual and movement fields, we collected data in a condition in which the monkey performed a delayed saccade task. During this task, the monkeys had to fixate the center of the screen while a saccade target was presented at one of 32 positions (4 amplitudes, 5, 10, 15 and 20 deg and 8 directions 4 cardinal directions and 4 oblique, covering the contralateral as well as the ipsilateral visual hemifield). After the appearance of the target, the monkey had to maintain fixation while the saccade target was presented for another 300–700 ms, after which the fixation point disappeared. The monkey then made a saccade to the target. After the saccade the monkey had to maintain fixation on the saccade target for another 300–500 ms to obtain the reward. The different positions of the target were randomly interleaved across trials, and 5 trials were collected for each condition.

### Data Analysis

#### Remapping task

The procedures used for analysis of the remapping data were written in MATLAB (The MathWorks Inc.). We calculated the baseline activity during steady fixation, using data from all trial types in a time window between 200 ms before and 20 ms after the onset of the visual probe and/or the saccade target. The peri-stimulus time histogram (PSTH) was calculated in a time-window between 30 ms and 550 ms after the onset of the probe by convolving each spike with a half-Gaussian (std = 30 ms) in which the values smaller than the mean were truncated. A half-Gaussian rather than a Gaussian was used to avoid biasing our estimates of response latency. The smoothed responses were then averaged across all trials. A response was considered significant (t-test, p<0.05) if it exhibited an increase of activity above the baseline for 30 consecutive bins of 1 ms duration each. The *latency* of the response was defined as the duration between the onset of the probe and the time when the activity became significant for the first time. For some analyses we used the same method to calculate the latency of the response relative to the onset of the saccade. The bimodality of the distribution of latencies was tested using Hartigan’s dip test of unimodality [24] in which the dip statistics of the latency distribution was compared with the dip statistics of a random, normally distributed sample.

One of the goals of our analysis was to distinguish between remapping responses that are typically observed in cortex and other types of perisaccadic changes in receptive field parameters, such as size or sensitivity. We refer to perisaccadic responses to stimuli placed outside of the receptive field defined during fixation as *extraclassical* responses. In our analyses, we considered an extraclassical response to have occurred when all of the following conditions applied: 1) a probe presented at the MID or the FF location in a saccade task elicited a significant increase in activity; 2) a probe presented at the MID or the FF location in a fixation task elicited no significant increase in activity; 3) a saccade without presentation of any visual probe caused no significant increase in activity. These criteria are similar to those used in previous work by other groups [8], [18–16]. The adoption of an additional criterion that the perisaccadic response at the FF position must be significantly stronger than that observed during fixation [10]–[25] yielded no difference in any of the main results of this study.

To quantify the strength of these responses, we calculated a continuous d’ value:
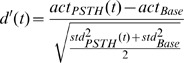
where *act_PSTH_(t)* is the peri-stimulus activity, *act_Base_* is the baseline activity, and *std_PSTH_(t)* and *std_Base_* are the inter-trial standard deviations of the peri-stimulus activity and the baseline activity. The maximal *d’* was used as an indicator of the detectability of the visual probe in the different experimental conditions.

#### Estimation of visual latencies for different probe luminances

As in the remapping task, latency was calculated as the duration between the onset of the probe and the first time the response became significant (p<0.05 in a time window of at least 30 ms). The average responses for each probe luminance were calculated only from the neurons that responded significantly to the respective luminance. Consequently the average latencies for probes with very low luminance were based on a much smaller sample of neurons than those for higher luminance probes.

#### Classification of neurons

We analyzed data from 136 neurons (64 from monkey 1, 72 from monkey 2) tested with the remapping paradigm. For comparison with previous literature, we also tested 96 of these neurons in the delayed saccade paradigm (described above), which allowed us to categorize them qualitatively as being either purely visual or visuo-motor, the latter having distinct visual and motor responses in the delayed saccade paradigm. Of these 96 neurons, 14 (15%) were classified as purely visual, and 82 (85%) were visuo-motor. Purely motor neurons were not tested.

Although we have not reconstructed our electrode tracks, it is likely that most of our recordings came from the intermediate layers of the superior colliculus. As mentioned above, we targeted these layers in most penetrations, and most of the neurons for which classification was possible were visuo-motor. The few purely visual cells included in the analysis were generally found at roughly the same depth as these visuo-motor neurons, despite the fact that most purely visual neurons are located in the superficial layers [26]. It is possible that some or all of these visual neurons were “quasivisual” cells [27], which can only be identified with certainty in a double-saccade task. Since the few purely visual neurons that showed significant responses to MID and FF probes did not show any other substantial differences from the rest of the population, we combined all neurons from the SC population for the analyses described below.

## Results

In this work we examined the perisaccadic responses of SC neurons to stimuli placed outside of their visual receptive fields. One important example of such an *extraclassical* response is the remapping of receptive field locations that occurs in many neurons around the time of a saccade. Another kind of extraclassical response is an apparent change in receptive field size, which can be brought about by a perisaccadic increase in visual sensitivity. To distinguish between these different types of extraclassical responses, we tested SC responses to visual probes placed in three different spatial locations.

### Responses to Stimuli Outside the Classical RF Around the Time of Saccades

We examined the responses of 221 neurons from the intermediate layers of the SC. From these 221 neurons, we eliminated 85 neurons that failed to show significant visual responses to probes flashed in the RF or that showed a significant increase in activity around the time of ipsiversive saccades in the absence of a visual probe. For the remaining 136 neurons, we measured the responses to individual visual probes during fixation and around the time of saccades. As in previous studies, the visual probes were small spots of light that were flashed briefly against a completely dark background. In order to assess the frequency of remapping, we compared the responses to probes presented in the receptive field (RF) and the future field (FF). The latter is defined as the RF position shifted by the vector of the impending saccade. As an additional point of comparison, we interleaved trials in which the probe was presented midway (MID probes) between the RF and the FF.

The activity for one example neuron is shown in Figure 2A. During fixation trials (first row) this neuron responded strongly to a probe presented in the RF (left panel) but not to probes presented in the MID (second panel), or FF (third panel) positions. When a visual probe was presented during saccade trials, (second row), the RF response remained, but in addition the neuron also responded to probes at the FF with a post-saccadic response occurring some 300 ms after the probe had been extinguished. In the third row the responses in the FF are shown aligned to the onset of the saccades. In control trials when no probe was presented, no increase in activity was observed in the fixation or saccade conditions (rows 1 and 2, right panels). Figure 2B shows a second neuron that responded only to probes at the RF position during fixation, but that had significant responses to MID and FF probes around the time of a saccade.

In order to quantify these discharge properties across the population, we defined significant “extraclassical” responses as those that were significantly (p<0.05, t-test) higher than the mean baseline firing rate in a time window of at least 30 ms duration. Using this criterion, 41 out of 136 neurons (30%) showed such responses to probes presented in the FF. This percentage is comparable to that reported by Walker et al. [11] who also reported that 30% of their SC neurons showed remapping responses. Furthermore, in our sample, 33 out of 136 neurons (24%) showed responses to probes presented at the MID position.

### Spatiotemporal Properties of Extraclassical Responses

In order to study the temporal profiles of extraclassical responses, we calculated the latencies of the responses to RF, MID and FF probes. The distributions of these latencies relative to probe onset are shown in Figures 3A and 3B. For probes presented in the RF during saccade trials, the latencies relative to probe presentation (X axis) are short and quite uniform (mean: 55 ms, std: 15 ms), while for probes presented at the MID and FF positions there is substantial variability. Closer examination of these latencies indicates a bimodal distribution for both probe locations, regardless of whether latency is calculated relative to probe onset (Figures 3A and 3B) or to saccade onset (Figures 3C and 3D). The short-latency responses (called *early* extraclassical responses in the rest of the paper) occur mainly around the time of saccade onset, while the distribution of long latency responses (*late* extraclassical responses) is centered around 150–200 ms after saccade onset. Each of the four latency distributions shown in Figure 3 differed significantly from unimodality (Hartigan’s test of unimodality, p<0.05).

To quantify these timing effects, we fit the latency distributions for the MID and FF positions with a sum of two Gaussians. These are shown in Figure 3 as red and blue curves; details of the distributions of early and late responses to MID and FF probes are summarized in Table 1. In order to objectively distinguish early from late extraclassical responses, we defined a border between the two latency distributions as the point of intersection of the two Gaussians. For responses relative to the onset of the visual probe this intersection was at 198 ms for probes at the MID position and 190 ms for probes at the FF position. Similar results were obtained when we used the latencies relative to the saccade onset where the intersections were at 90 ms for MID probes and 106 ms for FF probes. In some cases (5 for MID probes, 7 for FF probes) individual neurons were found to exhibit both response components, as seen for example in Figure 2B. For these neurons we treated the late response as separate from the early one if the two responses were separated by a period of at least 30 ms of activity that was not significantly above baseline. To verify that the differences in latencies of the two groups were genuine, in Figure 3 E and F we show the average normalized population activities for the neurons that exhibited only early responses (blue lines) and for those that exhibited only late responses (red lines) to MID probes (E) and FF probes (F). In both cases a clear increase in activity in the respective time window can be seen.

One possible explanation for early extraclassical responses is a perisaccadic increase in the size of collicular receptive fields similar to that found previously in the superficial layers of SC [28]. That is, what might appear as a remapping of visual space may actually result from an increase in perisaccadic sensitivity to stimuli placed at the fringes of the receptive fields. This is quite plausible, as previous work has shown increased sensitivity to weak visual stimuli around the time of a saccade [29]. Moreover, weak stimulation is generally associated with longer response latencies [30]. In this case early responses at both the FF and MID positions should have latencies similar to those obtained when weak stimuli are presented in the RF centers of SC neurons.

For the neurons in Figures 3 A and B, the early MID and FF responses have average latencies of ∼100 ms after the onset of the visual probe. These were significantly longer than the visual responses to RF probes (p<0.001, t-test) as summarized by the histograms at the top of panels A and B. To determine whether this latency increase was consistent with weak stimulation of the RF fringe, we measured the latency of neuronal responses to visual probes of different luminance presented at the RF position during continuous fixation and around the time of saccades. The results are shown in Figure 4 for probes presented during fixation (blue line) and before saccades (red line); in both cases the latencies increase from ∼50 ms for strong visual stimuli up to ∼150 ms for stimuli that were close to the threshold of detectability. Thus the typical latency of the early (but not of the late) responses in the remapping paradigm (green horizontal line in Figure 4) is well within the range of visual responses to weak stimuli presented within the RF. Moreover, we found that significant responses to probes at the MID position were significantly (p<0.05, χ^2^ test) more common when the MID position fell within the visual field contralateral to the SC from which we recorded (22/61; 36%) than when it fell within the ipsilateral visual field (5/30; 16%). In an additional 8/45 (18%) of the cases, responses to MID probes were found when the MID probe was presented vertically above or below the fixation point and therefore could not be assigned to a specific visual hemifield. Overall these results are consistent with a perisaccadic modulation of visual sensitivity.

### Comparison of Responses to MID and FF Probes

The previous section suggests that the early extraclassical responses - with average latencies relative to probe onset ∼100 ms - are consistent with an expansion of the classical receptive field around the time of a saccade. If that were true, one would expect to find that these responses were stronger for MID probes than for FF probes, as the former are closer to the center of the classical RF. To test this hypothesis, we assessed the response strength at the MID and FF positions for each neuron, using the maximal d’ values (see Methods for definition) as a measure of response strength. For this test we included only those neurons that showed a significant perisaccadic response to FF or MID probes in the absence of significant responses to these probes when they were presented during fixation. This left 32 neurons that showed early extraclassical responses and 29 neurons that showed late extraclassical responses. Figure 5 shows that a majority of the neurons with early responses exhibited significant responses only to probes presented at the MID position (17/32, 53%) (Figure 5A, red squares). In comparison, relatively few of these cells (6/32, 19%) showed significant responses to probes presented in the FF only (Figure 5A, blue circles). For neurons that showed significant responses to probes at both locations (Figure 5A, green stars; 9/32, 28%), the responses were stronger at the MID positions in 7 of 9 cases. This finding is consistent with the idea that significant early responses in the remapping paradigm are not due to remapping but rather to a perisaccadic increase of the RF size.

In contrast to the results obtained for early extraclassical responses, the late extraclassical responses (Figure 5B) were more consistent with the standard spatial profile of remapping. Of the population that exhibited significant late remapping, 8/29 (28%, red squares) neurons showed responses only to the MID probes, while 15/29 (52%, blue circles) showed significant responses only to the FF probes. The remaining cells (6/29, 21% green stars) showed remapping for probes at both positions; half of those cells showed stronger responses to the MID probes and the other half to the FF probes.

### Temporal Alignment of Early and Late Responses

Remapping has typically been associated with neural activity locked to saccade onset [16], as would be expected for a corollary discharge mechanism responsible for perisaccadic stability. On the other hand, our results regarding early extraclassical responses are consistent with a perisaccadic modulation of visual signals. These signals, being primarily visual in origin, would presumably be locked to probe onset.

One way to investigate the alignment of the responses is to relate the latencies of extraclassical responses to the saccadic reaction times (SRTs). For visual responses one would expect no relationship between the SRTs and the latency of the neuronal response, while if the responses were better aligned to the onset of the saccade, SRTs and remapping latencies should covary. There was a substantial difference between the SRTs of our two monkeys (mean 128 ms, std 16 ms in monkey 1, mean 215 ms, std 30 ms in monkey 2), which we used to investigate whether the latency of the neuronal responses changed accordingly. The comparison between SRTs and neuronal latencies for the two monkeys is shown in Figure 6. The differences in early response latencies (blue lines) are not significant between the two monkeys for MID probes (Figure 6A; p = 0.24, t-test) or for FF probes (Figure 6B; p = 0.21, t-test). In contrast, the differences between the latencies of the late responses (red lines) are significantly different in both cases (p<0.01 for MID probes, p<0.001 for FF probes; t-test), and monkey 2 showed longer neuronal as well as longer SRTs. The increases in latency of late remapping also quantitatively matched the differences in SRT between the two monkeys (dashed black lines in Figure 6). These results suggest that while the early responses are independent of the timing of the saccades, the timing of the late remapping is aligned to saccade onset.

## Discussion

We investigated perisaccadic remapping in the primate SC by recording single-neuron responses to probes presented at various times relative to saccades. Consistent with previous studies [11], we found significant responses to stimuli placed in the future receptive fields of many neurons. In addition, we found a bimodal distribution of latencies for these responses, which we term *early* and *late* extraclassical responses. Early extraclassical responses had latencies within the range of responses to weak stimuli inside the classic receptive field; they also occurred more often for stimuli placed midway between the receptive field and future field. These might therefore be associated with a perisaccadic increase in receptive field sensitivity, rather than a remapping of spatial positions. In contrast, late responses were more often associated with probes in the future field and had latencies that were correlated with saccade onset.

### Comparison to Previous Studies on Remapping in SC

Three earlier studies have described remapping in the intermediate layers of the SC. In the first study, Walker et al. [11] reported a bimodal distribution of response latencies for stimuli in the future field. While this bimodal distribution appears superficially similar to our findings (Figure 3), it actually reflects a very different set of experimental contingencies. While in our study the probe was flashed for 59 ms at the same time as the onset of the saccade target, the probe in the experiments by Walker et al. [11] was continuous and therefore present before, during and after the saccade. Thus the post-saccadic responses found by Walker et al. were not remapping but rather a purely visual ‘reafferent’ response to the appearance of the probe in the classical RF, as stated by the authors. These reafferent visual responses in all likelihood obscured the late remapping response that we describe here. In contrast the remapping responses reported by Walker et al. are most likely identical to our *early* extraclassical responses. In a second set of experiments Walker et al. also used a flashed probe similar to our approach. They found presaccadic remapping responses whose latencies relative to saccade onset were similar to those they obtained with a continuously lit probe. However, for this flashed probe condition, they did not describe post-saccadic responses with latencies that were much longer than saccade onset.

Dunn, Hall and Colby [13], using a flashed-probe paradigm similar to ours, also found that remapping latencies in the SC were highly variable. Although they did not report a bimodal distribution of latencies, they found many cells that exhibited ‘predictive remapping’, defined as a future field response that occurs with a latency (relative to saccade onset) that is shorter than that observed for a visual response to a probe presented in the RF. This predictive remapping appears to be very similar to the early extraclassical responses in our study. Dunn et al. [13] have further shown that only remapping responses with short latencies were reduced in split-brain monkeys; this effect was similar to observations made previously in LIP [9], which suggests a link between remapping processes in LIP and the intermediate layers of SC. This result also implies that there is a fundamental difference in the neuronal mechanisms that generate early and late extraclassical responses. Our results support this interpretation of the data.

In a recent study [12] we found that all remapping responses in the SC - early and late - are highly sensitive to the overall background luminance level. Indeed, although remapping was fairly common in complete darkness, such responses were usually decreased or abolished in the presence of modest background illumination. Given that the change in luminance affected both early and late remapping responses, it seems likely that the underlying mechanism resides within the SC itself.

### Different types of Extraclassical Responses?

Consistent with previous studies, we have found a wide range of latencies of extraclassical responses [8], [10]–[11], [14]–[18]. This variability may be due to a single mechanism that acts on different time scales or to a number of mechanisms, each with its own temporal profile.

There are several aspects of our results that favor an interpretation in terms of at least two distinct mechanisms. First, the distribution of latencies in our sample is truly bimodal, rather than simply being highly variable. The early latencies occur roughly 100 ms after onset of the visual probe while the late responses are centered ∼200 ms after the saccade onset. This pattern of results is unlikely to arise from a single process that generates both groups of latencies but none in between. As mentioned in the Results (Figure 4), the early latencies are within the range of visual latencies to weak visual probes flashed in the classical RF, while the longer latencies are much longer than visual latencies observed in response to RF probes.

Second, the temporal alignment of the activity is different for the early and the late responses. The early responses occur at a fixed time relative to the onset of the visual probe and are independent of saccadic latency, while the late responses occur at a fixed time relative to the onset of the saccade. To our knowledge the only study that explicitly investigated the alignment of remapping responses was conducted in the FEF [16], where remapping was shown to start at a fixed time relative to the onset of the saccade. The late extraclassical responses in SC therefore resemble remapping in the FEF, while early extraclassical responses behave more similarly to conventional visual responses.

Third, we observed differences in the spatial extent of early and late extraclassical responses. We found a substantial number of neurons that showed what appeared to be remapping responses but, that upon closer inspection, were actually stronger for stimuli placed midway between the receptive field and the future field. Although these responses were not standard visual responses, as they were not present during continuous fixation, they resembled visual responses in that they were stronger when the probes were presented closer to the center of the classic RF. Thus, these results might more parsimoniously be interpreted as a perisaccadic rescaling of the sensitivity of a fixed spatial RF, rather than a shift in RF position. Indeed, several other studies have reported changes in classical receptive fields around the time of saccades, including changes in sensitivity [31–29] and size [28], [32–17]. This finding may be unique to the SC, as a similar response to MID probes was not found for a small sample of FEF neurons [16]. In contrast, an increase in RF size does not account for cases when the extraclassical responses were stronger at the FF position than at the MID position (Figure 5B), as was the case in the majority of our late remapping neurons.

The claim that there are different types of extraclassical responses has also been made in several earlier studies based on the different latencies of remapping responses [8]–[14], [15]–[9]. Specifically, the ‘predictive visual responses’ and ‘memory responses’ observed in the previous work appear to be consistent with the early and late extraclassical responses described here. In the present study we have shown that these two types of responses have very different properties. The early responses resemble visual responses, in that they have similar latencies to the responses to weak visual stimuli that are flashed in the classical RF. Their latency is aligned to the onset of the visual probe rather than the onset of the saccade and they are often strongest at locations between the RF and the FF. In contrast, the late responses resemble memory traces of the stimulus that activate the neuron when they are placed inside the RF.

### Sources of Remapping in SC

A previous study by Dunn at al. [13] examined remapping responses following trans-section of the forebrain commissures, which disconnects the cortical hemispheres. This manipulation had similar effects on short-latency remapping in SC and LIP. On this basis Dunn et al. [13] proposed that one of the sources of remapping in SC is via the monosynaptic connection from LIP to SC [33], [34]–[35], [36]–[37]. Although we propose that there are different mechanisms behind the early and late extraclassical responses, it is still possible that both types have their origin in LIP. To confirm or reject this speculation one would need to investigate the spatial and temporal profiles of remapping activity in LIP to assess the similarity of remapping in the LIP and SC. In the work of Heiser at al. [9], the distribution of remapping latencies for LIP neurons around the time of contraversive saccades (their Figure 9B) appears somewhat bimodal, with a group of neurons having shorter latencies than the onset of the saccade and another group having latencies longer than ∼80 ms after the saccade onset. Therefore, at the first glance the timing of LIP remapping is similar to that of SC remapping, but a closer investigation of LIP remapping is required to confirm this speculation.

### Functions of Remapping

The differences in latency of the two groups of neurons suggest a differential involvement in perceptual and motor tasks. Remapping has been often discussed as a possible source of trans-saccadic perceptual stability (reviewed in [38]–[39]). This is consistent with what we describe as early extraclassical responses and with what is described elsewhere [9]–[13] as predictive remapping. In these cases remapping provides information about a visual stimulus more quickly than the normal reafferent visual responses, which helps to blend the presaccadic and the postsaccadic visual images and to decrease the latency of subsequent saccades. Indeed it has been shown that elimination of remapping in some cortical areas (FEF, [19], LIP, [9]) impairs performance on a double-saccade task. However, at least in SC the early remapping seems not to be spatially accurate, since probes presented midway between the RF and the FF often elicit stronger responses than FF probes.

A different function must be proposed for remapping with latencies of ∼200 ms after saccade onset. These responses may be relevant only in situations in which a stimulus that is present before a saccade disappears before the saccade onset or during the saccade as in the standard double-saccade task [40–19]. In cases when the presaccadic stimulus is still present after the saccade, it would generate normal reafferent activity, which would be a stronger and presumably more accurate source of information to guide a second saccade. If the secondary target disappeared just before or during the saccade, the remapping signal would represent a memory trace that would remain the only source of information that could be used for orientation towards the position of a behaviorally relevant presaccadic stimulus.
